# Supplementary material for: The Stat3-Fam3a axis promotes muscle stem cell myogenic lineage progression by inducing mitochondrial respiration
Source: Nat Commun. 2019 Apr 17;10:1796. doi: 10.1038/s41467-019-09746-1 (PMC6470137; doi:10.1038/s41467-019-09746-1)
Supplement: Supplementary file 1 — Supplementary Information [file 41467_2019_9746_MOESM1_ESM.pdf]

The Stat3-Fam3a axis promotes muscle stem cell myogenic lineage progression by inducing mitochondrial respiration

Sala et al.

Supplementary Figure 1

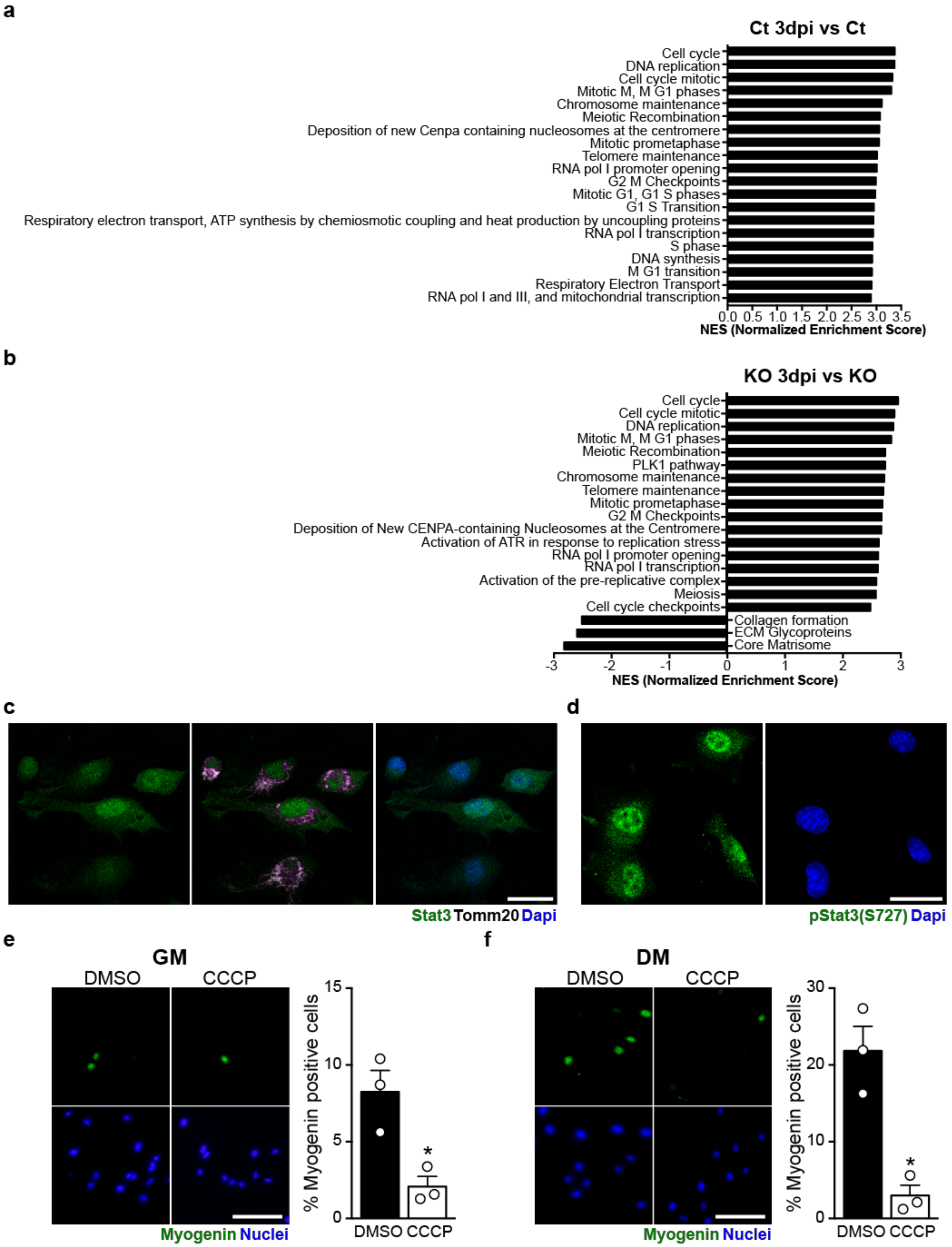

Supplementary Figure 1. Mitochondrial respiration is essential for proper MuSC myogenic commitment and differentiation.

(**a**) Top 20 enriched pathways (using GSEA) in freshly isolated MuSCs from 3dpi control mice (Pax7-CreER^{WT};Stat3$^{f/f}$) compared to MuSCs isolated from uninjured control mice.

(**b**) Top 20 enriched pathways in freshly isolated activated (3dpi) Stat3 KO MuSCs (Pax7-CreER;Stat3$^{f/f}$ mice) compared to uninjured Stat3 KO MuSCs by GSEA.

(**c-d**) Immunofluorescence analysis of the localization of total Stat3 and S727 phosphorylated Stat3 in MuSCs cultured in growth conditions for 72 hours. Scale bars 25 µm.

(**e**) Immunofluorescence analysis and quantification of myogenin expression of MuSCs cultured in growth conditions for 72 hours and treated with vehicle (DMSO) or CCCP (12.5µM) for the last 24 hours (n=3 independent experiments). Scale bar 100 µm.

(**f**) Immunofluorescence analysis and quantification of myogenin expression of MuSCs cultured in differentiation conditions for 48 hours and treated with vehicle (DMSO) or CCCP (5µM) for the last 24 hours (n=3 independent experiments). Scale bar 100 µm.

Data represented as mean ± SEM (Student's t-test; * $p<0.05$).

Supplementary Figure 2

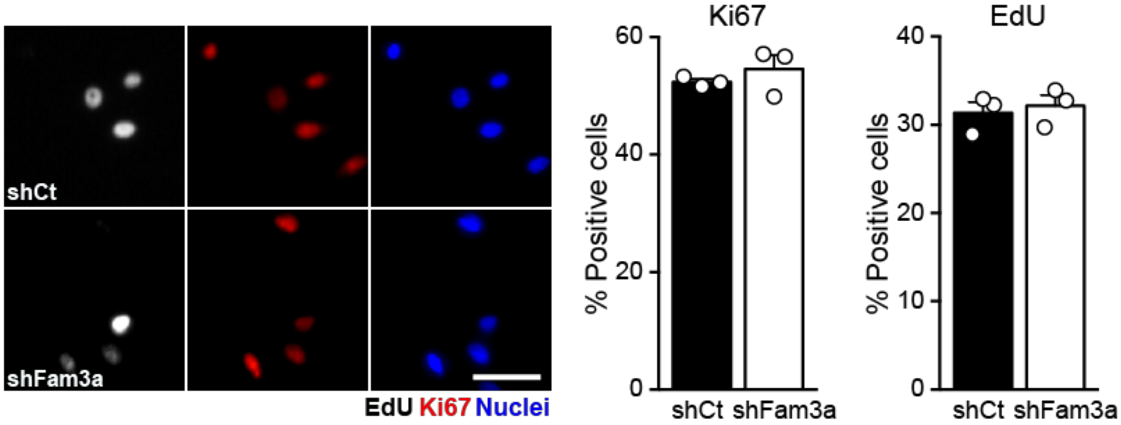

Supplementary Figure 2. Fam3a repression does not affect MuSC proliferation. Immunofluorescence analysis and quantification of proliferation of MuSCs infected with lentiviruses coding for an shRNA Control (shCt) or an shRNA against Fam3a (shFam3a), and cultured in growth conditions for 72 hours (n=3 independent experiments). Scale bar 50 μm. Data represented as mean ± SEM.

Supplementary Figure 3

a

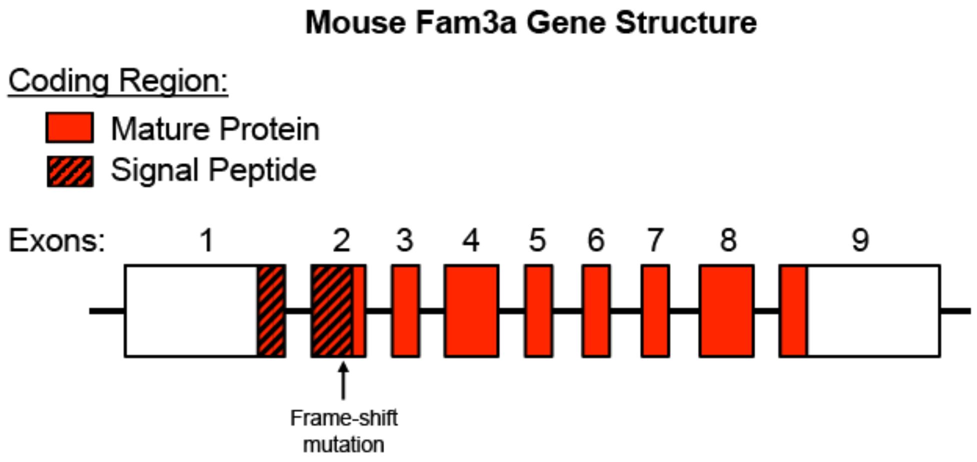

b

sgRNA target site + PAM sequence
Deleted bases

Fam3a WT allele
Part intron 2 + **Exon 2** + part intron 3

agtagaaggggagatctggtcatgtgaggtagtgggttattgcagattttgttcactgatgtctaagatagtcctgc
tctgtgtctatcctgcctgcag**GCCCCCTGCGCATCGTGGCCCTAATCATCATTATGGGTCTCACCTGGATCCTAGT**
CACCATCCTCCTAGGTGGTCCTGGTGTTGGCCTTCCTCGAATTCAGCAGTTCTTCACCAgtgagtgtgggaatcctc
ttgattcactat

Embryo 1 (Mosaic – we detected the presence of two different alleles by sequencing)

A1 (-8)
agtagaaggggagatctggtcatgtgaggtagtgggttattgcagattttgttcactgatgtctaagatagtcctgc
tctgtgtctatcctgcctgcag**GCCCCCTGCGCATCGTGGCCCTAATCATCATTATGGGTCTCACCTGGATCCTAGT**
CACCATCCTCCTAGGTGGTCCTGGTGTTGGCCTTCCTCGAATTCAGCAGTTCTTCACCAgtgagtgtgggaatcctc
ttgattcactat

A1' (-13)
agtagaaggggagatctggtcatgtgaggtagtgggttattgcagattttgttcactgatgtctaagatagtcctgc
tctgtgtctatcctgcctgcag**GCCCCCTGCGCATCGTGGCCCTAATCATCATTATGGGTCTCACCTGGATCCTAGT**
CACCATCCTCCTAGGTGGTCCTGGTGTTGGCCTTCCTCGAATTCAGCAGTTCTTCACCAgtgagtgtgggaatcctc
ttgattcactat

Embryo 2

A1 (-13)
agtagaaggggagatctggtcatgtgaggtagtgggttattgcagattttgttcactgatgtctaagatagtcctgc
tctgtgtctatcctgcctgcag**GCCCCCTGCGCATCGTGGCCCTAATCATCATTATGGGTCTCACCTGGATCCTAGT**
CACCATCCTCCTAGGTGGTCCTGGTGTTGGCCTTCCTCGAATTCAGCAGTTCTTCACCAgtgagtgtgggaatcctc
ttgattcactat

Embryo 3

A1 (-145)
agtagaaggggagatctggtcatgtgaggtagtgggttattgcagattttgttcactgatgtctaagatagtcctgc
tctgtgtctatcctgcctgcag**GCCCCCTGCGCATCGTGGCCCTAATCATCATTATGGGTCTCACCTGGATCCTAGT**
CACCATCCTCCTAGGTGGTCCTGGTGTTGGCCTTCCTCGAATTCAGCAGTTCTTCACCAgtgagtgtgggaatcctc
ttgattcactat

Embryo 4

A1 (-8)
agtagaaggggagatctggtcatgtgaggtagtgggttattgcagattttgttcactgatgtctaagatagtcctgc
tctgtgtctatcctgcctgcag**GCCCCCTGCGCATCGTGGCCCTAATCATCATTATGGGTCTCACCTGGATCCTAGT**
CACCATCCTCCTAGGTGGTCCTGGTGTTGGCCTTCCTCGAATTCAGCAGTTCTTCACCAgtgagtgtgggaatcctc
ttgattcactat

Embryo 5

A1 (-49)
agtagaaggggagatctggtcatgtgaggtagtgggttattgcagattttgttcactgatgtctaagatagtcctgc
tctgtgtctatcctgcctgcag**GCCCCCTGCGCATCGTGGCCCTAATCATCATTATGGGTCTCACCTGGATCCTAGT**
CACCATCCTCCTAGGTGGTCCTGGTGTTGGCCTTCCTCGAATTCAGCAGTTCTTCACCAgtgagtgtgggaatcctc
ttgattcactat

Pup 1

A1 (-10)
agtagaaggggagatctggtcatgtgaggtagtgggttattgcagattttgttcactgatgtctaagatagtcctgc
tctgtgtctatcctgcctgcag**GCCCCCTGCGCATCGTGGCCCTAATCATCATTATGGGTCTCACCTGGATCCTAGT**
CACCATCCTCCTAGGTGGTCCTGGTGTTGGCCTTCCTCGAATTCAGCAGTTCTTCACCAgtgagtgtgggaatcctc
ttgattcactat

Pup 2

A1 (-1)
agtagaaggggagatctggtcatgtgaggtagtgggttattgcagattttgttcactgatgtctaagatagtcctgc
tctgtgtctatcctgcctgcag**GCCCCCTGCGCATCGTGGCCCTAATCATCATTATGGGTCTCACCTGGATCCTAGT**
CACCATCCTCCTAGGTGGTCCTGGTGTTGGCCTTCCTCGAATTCAGCAGTTCTTCACCAgtgagtgtgggaatcctc
ttgattcactat

Pup 3

A1 (-13)

agtagaaggggagatctggtcatgtgaggtagtgggttattgcagattttgttcactgatgtctaagatagtcctgc
tctgtgtctatcctgcctgcag**GCCCCCTGCGCATCGTGGCCCTAATCATCATTATGGGTCTCACCTGGATCCTAGT**
CACCATCCTCCTAGGTGGTCCTGGTGTTGGCCTTCCTCGAATTCAGCAGTTCTTCACCAgtgagtgtgggaatcctc
ttgattcactat

Adult mice

A1 (-4)
agtagaaggggagatctggtcatgtgaggtagtgggttattgcagattttgttcactgatgtctaagatagtcctgc
tctgtgtctatcctgcctgcag**GCCCCCTGCGCATCGTGGCCCTAATCATCATTATGGGTCTCACCTGGATCCTAGT**
CACCATCCTCCTAGGTGGTCCTGGTGTTGGCCTTCCTCGAATTCAGCAGTTCTTCACCAgtgagtgtgggaatcctc
ttgattcactat

Supplementary Figure 3. Genotype information of the E15 Embryos, P0 pups, and adult mice used in this study.

(**a**) Scheme of the mouse Fam3a gene structure showing the point where the frame-shift mutations were induced.

(**b**) DNA sequences of the Fam3a WT allele and the Fam3a mutated alleles of the 5 Fam3a KO embryos, the 3 Fam3a KO P0 pups, and the adult mice used in this study. The sgRNA sequence, PAM sequence and deleted bases are shown.

Supplementary Figure 4

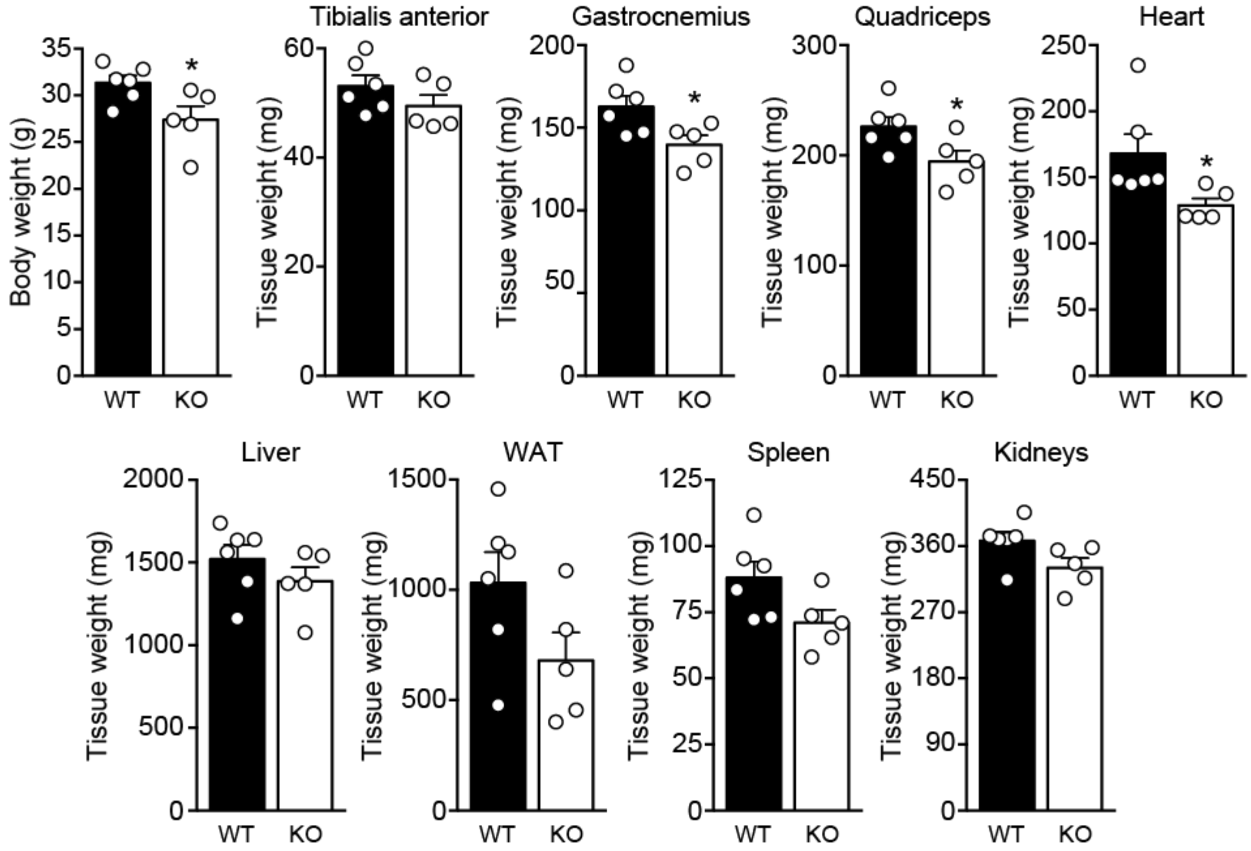

Supplementary Figure 4. Fam3a KO adult mice display reduced skeletal and heart muscle mass. Body and tissue weights from 3-month-old WT and Fam3a KO male mice (n=5-6 animals).

Data represented as mean ± SEM (Student's t-test; * $p<0.05$).

Supplementary Figure 5

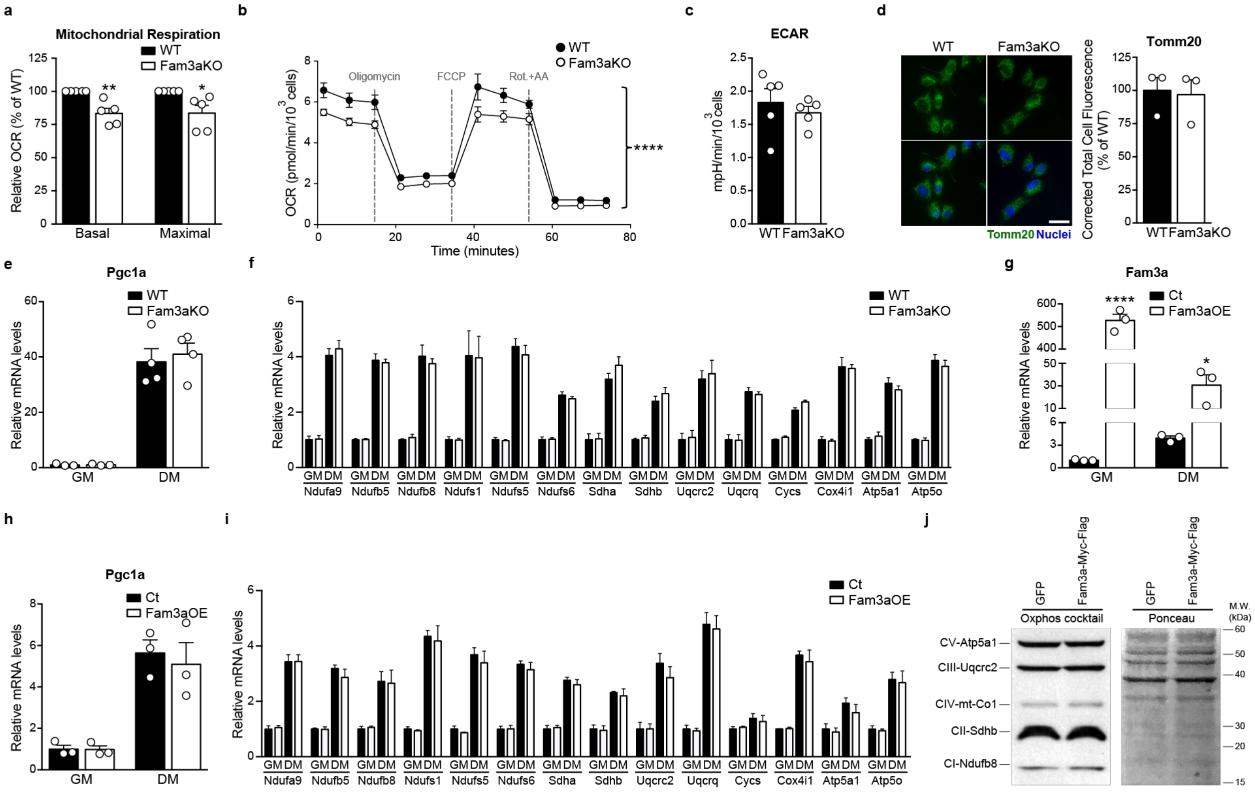

Supplementary Figure 5. Fam3a does not promote mitochondrial respiration by regulating mitochondrial content or the expression of the electron transport chain complexes.

(**a-c**) Measurement of the Oxygen Consumption Rate (OCR) and the Extracellular Acidification Rate (ECAR) of WT and Fam3a KO MuSCs cultured in growth conditions for 3 days (n=5 independent experiments). The Cell Mito Stress Test was performed using the Seahorse XFp Analyzer.

(**d**) Immunofluorescence analysis of the mitochondrial content in WT and Fam3a KO MuSCs cultured in growth conditions for 3 days. Tomm20’s corrected total cell fluorescence was quantified (n=3 independent experiments; more than 250 cells per condition were quantified).

(**e-f**) Gene expression analysis of WT and Fam3a KO MuSCs cultured in Growth Media (GM) or Differentiation Media (DM) for 72 hours (n=3-4 independent experiments).

(**g-i**) Gene expression analysis of C2C12 myogenic cells transfected with a construct to overexpress Fam3a-Myc-Flag and cultured in growth media (GM) or differentiation media (DM) (n=3 independent experiments).

(**j**) Western blot analysis of C2C12s transfected with constructs to overexpress GFP or Fam3a-Myc-Flag.

Data represented as mean ± SEM (Student's t-test or Two-way ANOVA; * $p<0.05$, ** $p<0.01$, **** $p<0.0001$).

Supplementary Figure 6

a

LocTree3

Protein ID	Score	Expected Accuracy	Localization Class
Fam3a_Mouse	23	84%	Secreted

TargetP

Name	Len	mTP	SP	Other	Loc	RC
Fam3a_Mouse	230	0.012	0.978	0.044	S	1
Citrate Synthase_Mouse	464	0.743	0.083	0.172	M	3

b

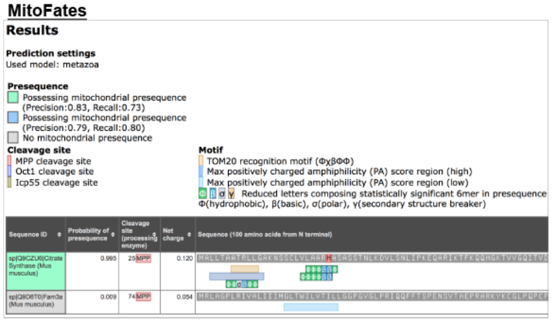

c

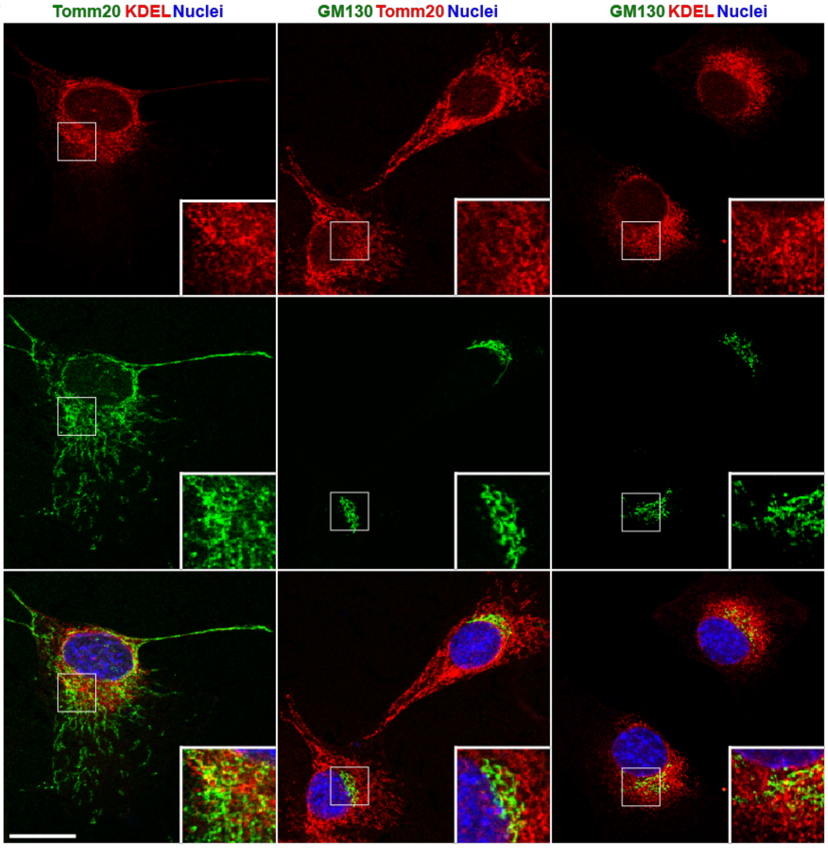

e

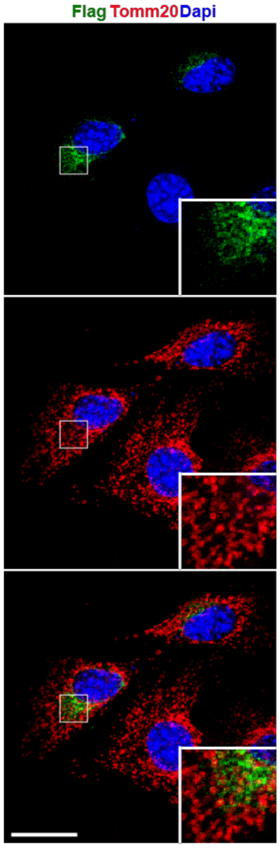

d

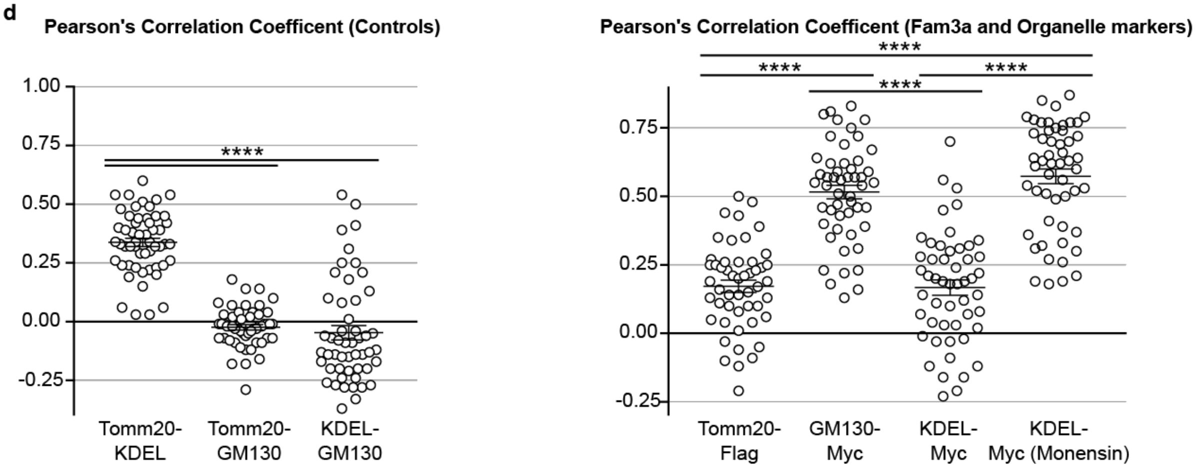

Supplementary Figure 6. Fam3a is a secreted protein.

(**a-b**) Fam3a localization predictions based on protein sequence and using LocTree3, TargetP and MitoFates softwares.

(**c-e**) Immunofluorescence analysis of C2C12 myogenic cells transfected with a construct to overexpress Fam3a-Myc-Flag. Tomm20 is a mitochondria marker, GM-130 is a cis-Golgi marker, and KDEL is an ER marker. (**c**) Representative images of the validation of the specific cell compartment labeling with the indicated markers. Yellow color means colocalization. (**d**) Quantification of the colocalization by analyzing the Pearson's Correlation Coefficient ($n \geq 50$ cells quantified per comparison). (**e**) Representative images for the comparison to assess the colocalization between Fam3a and mitochondria. Yellow color shows colocalization. Scale bars 20 μm.

Data represented as mean ± SEM (One-way ANOVA; **** $p<0.0001$).

Supplementary Figure 7

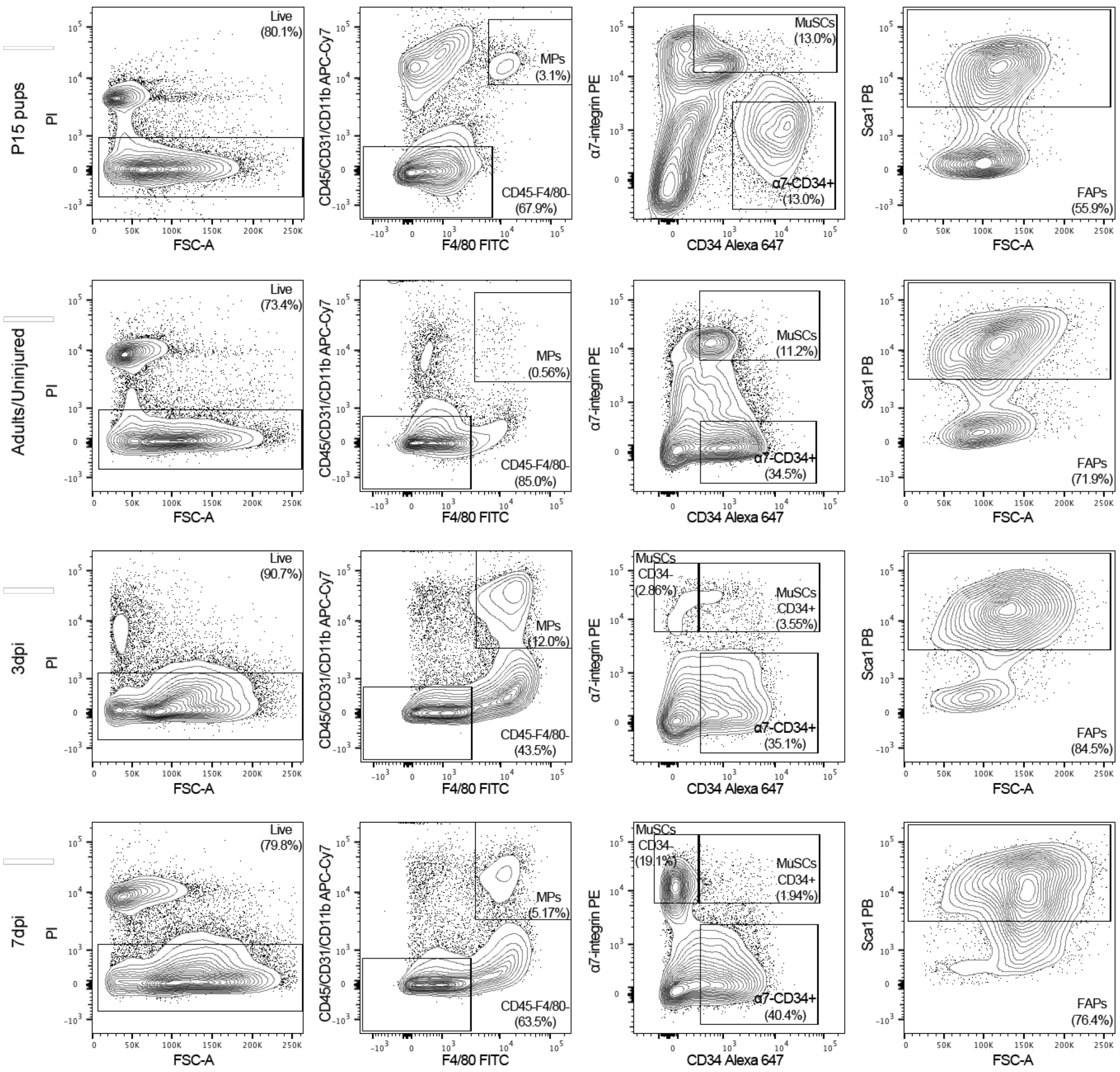

Supplementary Figure 7. Representative FACS plots and gating strategies for the isolation of mononucleated cells from hind limb skeletal muscles. P15 pups, and uninjured, 3dpi and 7dpi 3-month-old male mice were used. MuSCs were isolated as $CD45^{-}$ $CD11b^{-}CD31^{-}Sca1^{-}\alpha7\text{-integrin}^{+}CD34^{+}$ cells and used for RNAseq transcriptomic analysis (Fig. 1a-d; Fig. 2a; Supplementary Figure 1a-b), gene expression analysis (Figure 2b-c, Figure 5 f-g), and in vitro studies (Figure 1e-g; Figure 2d and h; Figure 3; Figure 6a-e; Supplementary Figure 1c-f; Supplementary Figure 2; Supplementary Figure 5a-f; Supplementary Figure 8). MuSC-

derived myogenic progenitors from 3dpi and 7dpi injured muscle were isolated as $CD45^{-}CD11b^{-}CD31^{-}Sca1^{-}\alpha7\text{-integrin}^{+}CD34^{-}$ cells and used for gene expression analysis (Figure 5g). Freshly isolated FAPs ($CD45^{-}CD11b^{-}CD31^{-}Sca1^{+}\alpha7\text{-integrin}^{-}CD34^{+}$) and MPs ($CD45^{+}CD11b^{+}CD31^{+}F4/80^{+}$) were used for gene expression analysis (Figure 5f-g).

Supplementary Figure 8

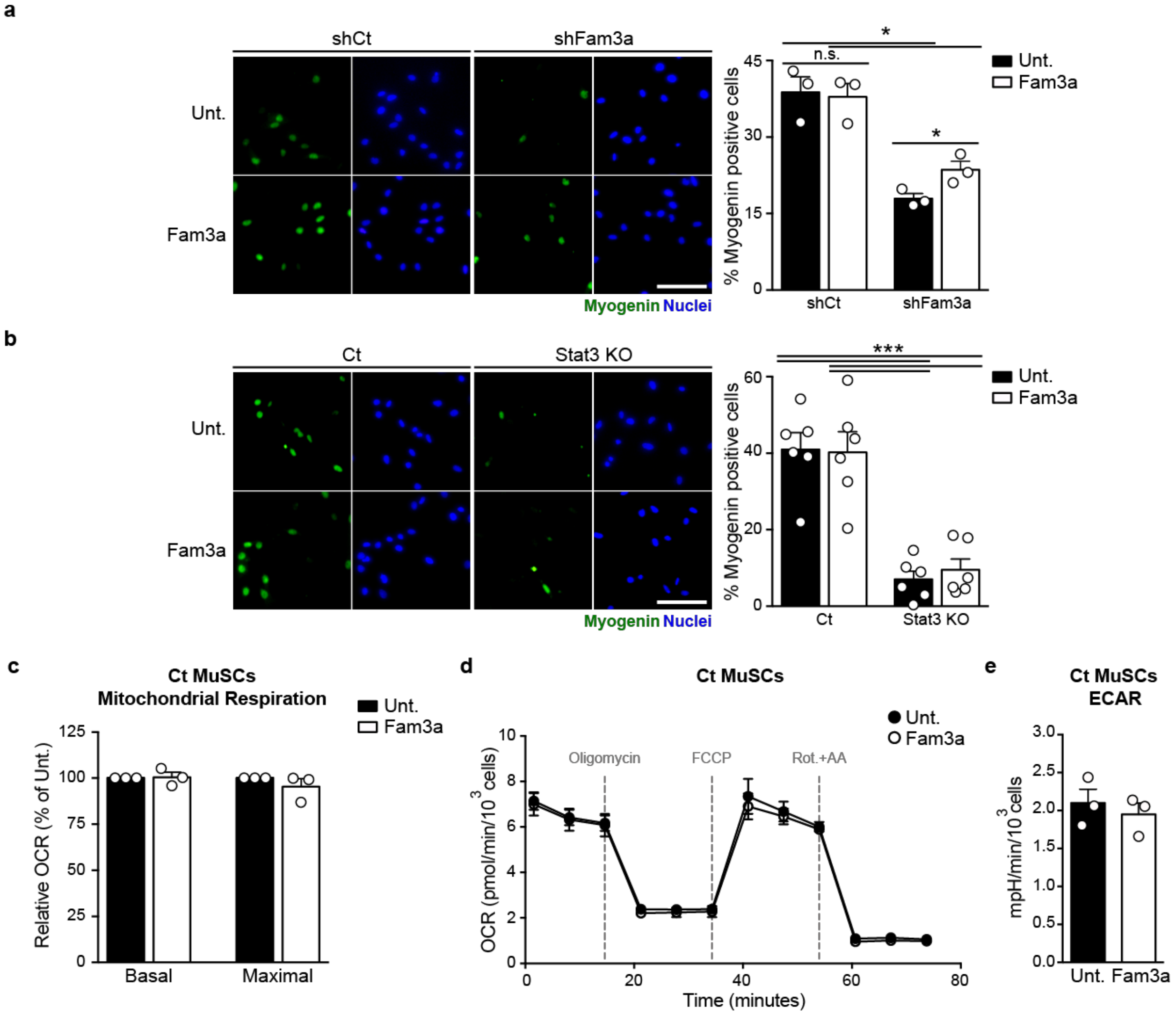

Supplementary Figure 8. Recombinant Fam3a rescues the impaired myogenic lineage progression of shFam3a MuSCs cultured in differentiation media.

(**a**) Immunofluorescence analysis and quantification of myogenin expression in MuSCs infected with shCt or shFam3a coding lentiviruses and cultured in differentiation conditions for 72 hours in the presence or absence of recombinant Fam3a (1000ng/ml) (n=3 independent experiments). Scale bar 100 μm.

(**b**) Immunofluorescence analysis and quantification of myogenin expression in control and Stat3 KO MuSCs cultured in differentiation conditions for 72 hours in the presence or absence of recombinant Fam3a (1000ng/ml) (n=6 independent experiments). Scale bar 100 μm.

(**c-e**) Measurement of the Oxygen Consumption Rate (OCR) and the Extracellular Acidification Rate (ECAR) of control MuSCs cultured in growth conditions for 3 days in the presence or absence of recombinant Fam3a (1000ng/ml) (n=3 independent experiments). The Cell Mito Stress Test was performed using the Seahorse XFp Analyzer.

Data represented as mean ± SEM (Student's t-test; * $p<0.05$, *** $p<0.001$).

Supplementary Figure 9

Figure 5c

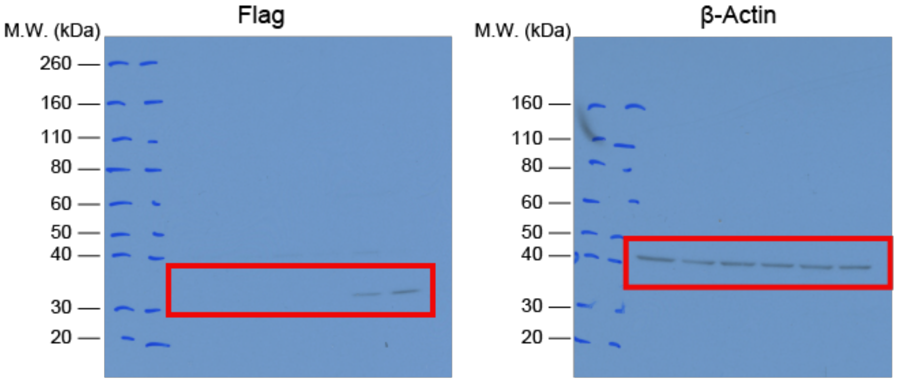

Figure 5e

Supplementary Figure 5j

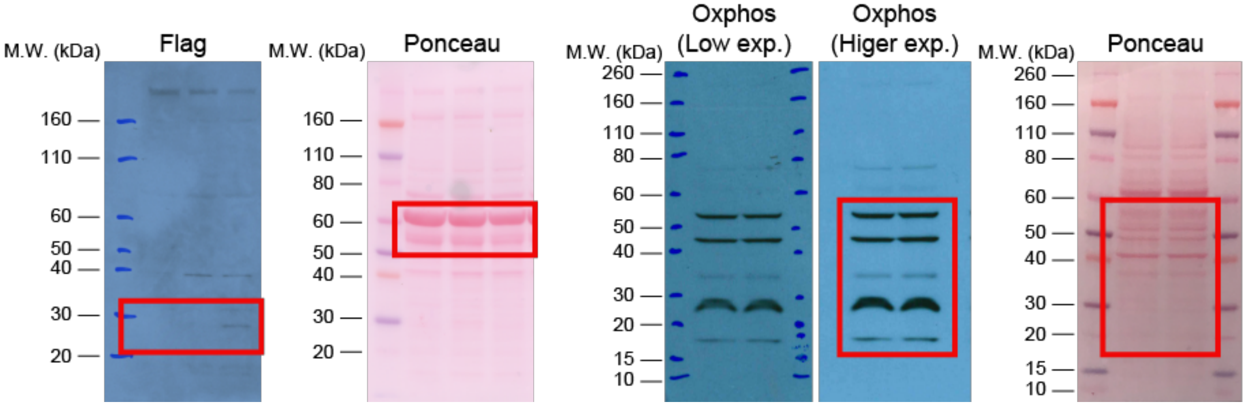

Supplementary Figure 9. Unprocessed scans for the Western Blots included in the manuscript.

Supplementary Table 1. Primers used in this study.

**Exon2 Genotyping Primers**	
Fam3aExon2_Fw	TGCTGAGGTTGGTGCTATGA
Fam3aExon2_Rv	AGTCACTGAGTTCTCTGGGC
**qPCR Primers**	
Rplp0_Fw	GCAGGTGTTTGACAACGGCAG
Rplp0_Rv	GATGATGGAGTGTGGCACCGA
BAct_Fw	GGTCATCACTATTGGCAACGA
BAct_Rv	GTCAGCAATGCCTGG
Fam3a_Fw	GGCCCTAATCATCATTATGGGTC
Fam3a_Rv	TGCAGTCACTGAGTTCTCTGG
Slc35b3_Fw	TCCGTATTTGGCCTAATCGAGC
Slc35b3_Rv	GCCCATAGTACCCACAGTTAGAA
Rgs20_Fw	AGAGCCTCCCATGAAATCAGA
Rgs20_Rv	GCTGGAGTGACCATCAGGTTAT
p19_Fw	GCCGCACCGGAATCCT
p19_Rv	TTGAGCAGAAGAGCTGCTACG
Ube2v1_Fw	AGGCTCGGGAGTAAAAGTCC
Ube2v1_Rv	GACCCCAGCTAACTGTGCC
Serp2_Fw	AGGGGAAACGTAGCCAAAACC
Serp2_Rv	GCCCATCCTTATGCTCTGTATG
Pard6a_Fw	TTTGACGCCGAGTTCCGAC
Pard6a_Rv	AGCATCCGTATAGCCAAGCAG
Aph1a_Fw	TGCTGTGTTTTTCGGATGCAC
Aph1a_Rv	TCTGATCGGTCTGTCACATGG
Akt1s1_Fw	CCCTGCTCCTAGTCCACCA
Akt1s1_Rv	TGTCTCTGTTTCAGTGGGCTC
Glo1_Fw	GATTTGGTCACATTGGGATTGC
Glo1_Rv	TCCTTTCATTTTCCCGTCATCAG
Pax7_Fw	ATGTTCAGCTGGGAAATCCGGG
Pax7_Rev	TCCCGAACTTGATTCTGAGCACTCG
Myod1_Fw	CCACTCCGGGACATAGACTTG
Myod1_Rv	AAAAGCGCAGGTCTGGTGAG
Myogenin_Fw	CAGTGAATGCAACTCCCACAG
Myogenin_Rv	ATGGACGTAAGGGAGTGCAGA
Stat3_Fw	TGAAGGTGGTGGAGAACCTC
Stat3_Rv	GCTGCTGCATCTTCTGTCTG
Ndufa9_Fw	GTCCGCTTTCGGGTTGTTAGA
Ndufa9_Rv	CCTCCTTTCCCGTGAGGTA
Sdha_Fw	GGAACACTCCAAAAACAGACCT
Sdha_Rv	CCACCACTGGGTATTGAGTAGAA
Sdhb_Fw	CTGAATAAGTGCGGACCTATGG
Sdhb_Rv	AGTATTGCCTCCGTTGATGTTC
Ndufb8_Fw	TGTTGCCGGGGTCATATCCTA
Ndufb8_Rv	AGCATCGGGTAGTCGCCATA
Ndufs6_Fw	GGGGAAAAGATCACGCATACC

Ndufs6_Rv	CAAAACGAACCCTCCTGTAGTC
Uqcrc2_Fw	AAAGTTGCCCCGAAGGTTAAA
Uqcrc2_Rv	GAGCATAGTTTTCCAGAGAAGCA
Cox4i1_Fw	TCCCCACTTACGCTGATCG
Cox4i1_Rv	GATGCGGTACAACTGAACTTTCT
Atp5a1_Fw	TCTCCATGCCTCTAACACTCG
Atp5a1_Rv	CCAGGTCAACAGACGTGTCAG
Ndufs1_Fw	AGGATATGTTCGCACAACTGG
Ndufs1_Rv	TCATGGTAACAGAATCGAGGGA
Uqcrq_Fw	CCTACAGCTTGTCGCCCTTT
Uqcrq_Rv	GATCAGGTAGACCACTACAAACG
Pgc1a_Fw	TGATGTGAATGACTTGGATACAGACA
Pgc1a_Rv	GCTCATTGTTGTACTGGTTGGATATG
Ndufb5_Fw	ACGCTCGCTTCTTGAGGTTA
Ndufb5_Rv	CTTCTGGGATTTCTGCAAGC
Cycs_Fw	GGGAGGCAAGCATAAGACTG
Cycs_Rv	CCAGGTGATGCCTTTGTTCT
Atp5o_Fw	GCAACACCCAGGGTATCATC
Atp5o_Rv	TTGGTTTGGACTCAGGAAGC
Ndufs5_Fw	GACATACAGAAAAAGCTGGGCA
Ndufs5_Rv	TCGCCTCATCGTTTTGTACCG
ChIP_Stat3BS_Fw	AGAGGTTTGGTCTGAAGAGATTT
ChIP_Stat3BS_Rv	CGAGCTGGAGTTACAGATGTT
