# Supplementary material for: The Stat3-Fam3a axis promotes muscle stem cell myogenic lineage progression by inducing mitochondrial respiration
Source: Nat Commun. 2019 Apr 17;10:1796. doi: 10.1038/s41467-019-09746-1 (PMC6470137; doi:10.1038/s41467-019-09746-1)
Supplement: Supplementary file 2 — Description of Additional Supplementary Files [file 41467_2019_9746_MOESM2_ESM.docx]

**Title:** Supplementary Data 1.
**Description:** Differentially enriched pathways in 3dpi Ct MuSCs vs Uninjured Ct MuSCs (GSEA).

**Title:** Supplementary Data 2.
**Description:** Differentially enriched pathways in 3dpi KO MuSCs vs Uninjured KO MuSCs (GSEA).

**Title:** Supplementary Data 3.
**Description:** Differentially enriched pathways in 3dpi KO MuSCs vs 3dpi Ct MuSCs (GSEA).
